# Affordable Motion Tracking System for Intuitive Programming of Industrial Robots

**DOI:** 10.3390/s22134962

**Published:** 2022-06-30

**Authors:** Martin Švejda, Martin Goubej, Arnold Jáger, Jan Reitinger, Ondřej Severa

**Affiliations:** NTIS Research Centre, Faculty of Applied Sciences, University of West Bohemia, 30100 Pilsen, Czech Republic; msvejda@ntis.zcu.cz (M.Š.); arnie87@ntis.zcu.cz (A.J.); reitinge@kky.zcu.cz (J.R.); osevera@ntis.zcu.cz (O.S.)

**Keywords:** industrial robots, motion tracking, pose estimation, trajectory planning, lead-through programming, collaborative robotics, HTC Vive, motion control, spray coating

## Abstract

The paper deals with a lead-through method of programming for industrial robots. The goal is to automatically reproduce 6DoF trajectories of a tool wielded by a human operator demonstrating a motion task. We present a novel motion-tracking system built around the HTC Vive pose estimation system. Our solution allows complete automation of the robot teaching process. Specific algorithmic issues of system calibration and motion data post-processing are also discussed, constituting the paper’s theoretical contribution. The motion tracking system is successfully deployed in a pilot application of robot-assisted spray painting.

## 1. Introduction

Robotic manipulators have become a key enabling technology in many domains of industrial automation. According to the International Federation of Robotics, an estimated 2.7 million industrial robots were in operation worldwide in the year 2020, with a sustained growth of future installations being predicted in the forthcoming years [1].

Robotic arms are typically employed in large production facilities with a high level of automation, such as automotive factories. However, they are readily becoming affordable for small to medium enterprises due to constant technological and economic development [2]. A common approach of one-time installation, commissioning, and programming by robotic specialists becomes inefficient for flexible automation tasks. Small series production inherently requires frequent changes in manufacturing operations. It introduces the demand for seamless reconfiguration of the robot that can be performed even by operating personnel without a deep background in robotics. Rapid development of collaborative robots addressing such needs can be observed in recent years [3]. Robots are breaking out of their cages on the factory floor and gradually become integrated into the manufacturing process with increasing mobility and interactivity with human workers [4].

One of the critical issues of collaborative robotics is the possibility of the simple transfer of skills necessary for an intended task from a human to a robot. Experienced operators with a deep knowledge of the involved process to be automated are seldom experts in robotics and vice-versa. Producers of robotic systems introduce novel approaches to intuitive programming without the necessity of traditional hand-coding in a robot-specific language. An example of this trend is in various hand-guidance systems for robots allowing simple interactive reconfiguration of the robot without a tedious jogging procedure using a teach-pendant [5,6].

The hand-guidance systems are typically used to position the robot interactively between a set of discrete pose configurations in its workspace. The points of interest are stored in a memory and interconnected by predefined motion commands. While this may serve well for simple jobs such as pick-and-place, continuous motion capture of a human demonstrating the task to be learned by the robot may be required in more complex applications. This approach, sometimes designated as “lead-through” programming, allows a considerable reduction of robot commissioning time when compared to traditional teach-pendant or offline coding methods [7,8]. It is a most natural way of teaching motion tasks requiring continuous pathways for most human operators, allowing simple demonstration of motion trajectories in the same manner as how a human would perform the task [9]. On the other hand, improper size, weight or shape of the robot often make the demonstration of the task difficult. The reason for that is that the human operator cannot entirely focus on the demonstrated task since he deals with the repositioning of the robot at the same time. This can be a source of various inaccuracies made during the demonstration that is hard to correct later.

The above-mentioned issues of the lead-through method led us to develop a motion-tracking device intended for simple and intuitive programming of industrial robots that would mitigate most of the inconvenience caused by traditional robot programming techniques. We started with a survey of existing tracking systems, focusing on their operating principles, achievable performance, and purchase cost.

Laser systems utilise an actively controlled source of laser beam that tracks one or more reflectors (markers) [10,11,12,13]. They are primarily intended for shape and dimension checking of manufactured parts during quality control. The accuracy is in the order of one to a few dozens of micrometres. However, some of them lack the capability of continuous motion tracking with a sufficiently fast update rate. A very high price (roughly 300 thousand to 3 million EUR) disqualifies them for our purpose.

Camera-based systems use one or more cameras and a set of active or passive markers. Their primary use is in the “Motion capture” applications, such as cinematography, motion analysis in medicine or research in drone control and robotics [14,15,16,17,18,19]. The price tag is substantially lower, ranging from 20 to 200 thousand EUR, based on the number of system components and achievable accuracy is in the order of one millimetre for continuous dynamic motion tracking.

Optical systems use either tracking of light markers in the visible or infrared spectrum or detection of swept light beams by a photo-sensor [20,21,22]. The pose estimation accuracy is several millimetres in translation and lower units of degrees in the orientation. The price is in the hundreds to thousands EUR, again varying with specific tracker configuration. Affordable price and reasonable tracking performance make this group viable for our purpose.

Alternative approaches using non-optical operating principles involve wearable inertial systems for human arm motion tracking [23], wire-based tracking devices using cable position encoders [24], or special position-sensitive detectors [25]. While some deliver sufficient tracking accuracy at a reasonable cost, they rely on complicated auxiliary mechanical devices that compromise potential flexibility and ease of use for the human operator.

The primary goal of this paper is to document the results achieved during the development of our motion tracking system for industrial robots. We present a solution built around the HTC Vive Tracker [26] and demonstrate its employment in a pilot application of robot-assisted spray coating, specifically addressing the following objectives:

- Objective 1—Describe the individual HW and SW components of the system. This may serve as an inspiration for readers from academia or industry working on similar topics.
- Objective 2—Develop generic algorithms for motion tracker calibration and recorded trajectory post-processing. These results are transferable to analogous motion control problems in various mechatronics applications.
- Objective 3—Evaluation of motion tracking performance in static and dynamic conditions. A fundamental step validating that the design specifications were met and the system is suitable for the intended purpose.
- Objective 4—Presentation of a pilot application in the field of spray coating. This serves as an industrial use-case demonstrating practical applicability of the proposed solution.

The paper is organised as follows. Section 2 introduces a high-level conception of the developed system, explaining the functionality and interconnection of the individual HW and SW components. Section 3 and Section 4 deal with specific details of the hardware and software modules, which constitutes the Objective 1. Section 5 describes important algorithmic details, addressing the Objective 2. Experimental results are shown in Section 6, focusing on validation of tracking system performance formulated in Objective 3 and pilot application from Objective 4. Concluding remarks and topics for future research are given in Section 7.

## 2. Overall Conception of the Motion Tracking System

**Figure 1:**
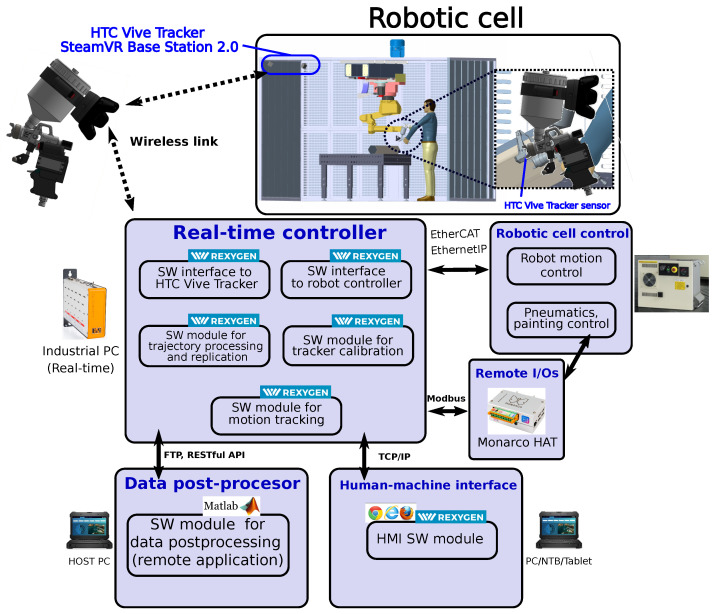
Overall conception of a robotic cell equipped with the motion tracking system.

The developed motion tracking system consists of several hardware and software modules interacting with each other via standardised communication interfaces. The overall structure and interconnection of the individual components are shown in Figure 1.

The main functionality of the developed tracking system is to accurately reproduce 6DoF motions of a tool held by a human operator by means of a robot arm. The usual workflow consists of the following steps:

1. The operator activates the tracking system and demonstrates the intended motion manually using a tool with attached tracker sensor.
2. Both absolute position and orientation are recorded in real-time for further processing.
3. The recorded motion trajectory is post-processed to smooth out measurement noise and hand-induced vibrations. Feed rate of the reproduced motions can also be adjusted as desired. The adjusted motions are uploaded back to the master controller.
4. The tool is attached to the robot arm and the device is switched to automatic mode.
5. The real-time controller commands the robot arm to reproduce the recorded trajectories in a fully automatic manner.

The HW part involves the motion sensor, industrial PC serving as a real-time controller, add-on input/output peripherals and communication interfaces. The SW modules implement methods and algorithms responsible for motion tracking, real-time trajectory recording, post-processing and replication via an employed robotic arm manipulator. A detailed description of the HW and SW components follows in the next sections.

## 3. Hardware Parts Specifications and Functional Properties

We had many discussions with industrial partners working in the field of robotics from which we identified several key features that a tracking system for robotic applications should have:

- Capability of recording of motion trajectory of a tool wielded by a human operator demonstrating a task, without the necessity to reposition the robot arm directly, followed by automatic reproduction of the motion task
- Tracking of the complete tool pose—both translation and orientation degrees of freedom are to be considered
- Possibility of attachment of the tracker sensor to a real tool used by a human operator to achieve maximum fidelity of motion demonstration and reproduction
- Affordable price of the tracking device, relatively low with respect to the price of the robot arm
- Robustness of the system allowing its employment in harsh industrial environments
- Compatibility with conventional industrial robots without a necessity of custom-made manipulators or mechatronic devices
- Self-contained system working as an add-on to the existing robot controllers
- Open architecture of the system allowing flexible customisation for different application fields
- Pose estimation accuracy of approximately 10 mm/1${}^{{}^{\circ}}$ for static/low-velocity motions and 30 mm/5${}^{{}^{\circ}}$ during dynamic tracking-suffices for applications with moderate precision requirements such as painting and coating, blasting, or handling and picking

Based on the performed survey of existing tracking devices, a solution based on the HTC Vive Tracker [26] was ultimately pursued due to the following reasons:

- Affordable price (approx. 500 EUR for the configuration with two base stations)
- Reasonable real-time tracking precision—submillimeter range for static/slow-motion position and <0.1${}^{{}^{\circ}}\;3\sigma$ attitude estimation errors, with an increase of factor 10 to 50 during dynamic motions [27,28], which is sufficient for moderately demanding applications of industrial robots
- Small dimensions (fits in 100 mm diameter sphere) and low weight (270 g) of the tracking sensor allowing simple attachment to the tracked tool without severely obstructing the motion of the operator during the teaching phase
- Commercial of-the-shelf product with long-term support from the manufacturer

### 3.1. HTC Vive Tracker

**Figure 2:**
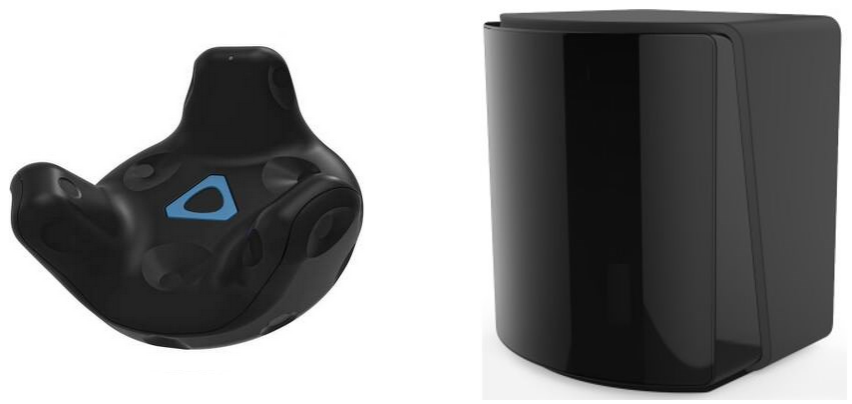
HTC Vive Tracker sensor (**left**) and base station (**right**).

HTC Vive is a cost-effective tracking system allowing real-time collection of ground truth position and attitude data. Its operating principle is based on infrared LEDs emitting synchronised light pulses from the lighthouses (base stations) and trackers that use photodiodes to measure light sweeps timings (Figure 2). The pose of the tracked devices can be reconstructed from the observed time differences. The sensor also contains an Inertial measurement unit (IMU) and performs data fusion with the optical measurements internally to enhance the precision of pose estimation.

The tracker is complemented by SteamVR Base Station 2.0. It is possible to install 1–4 stations at the same time, affecting achievable pose estimation accuracy (Figure 2 right). Data fusion and tracker calibration (relative pose to base stations that can be adjusted) is done internally directly in the tracker firmware. Modification of raw data processing algorithms is also possible when needed to fine-tune the tracker response in terms of sensor lag, static and dynamic accuracy. Software development support allows integration of the tracker system to 3rd party products. Wireless data transmission to a supervisory system can be established using a USB dongle. Easy assembly to the tracked device is achieved thanks to a standard tripod screw. Pogo pin connector at the bottom of the tracker allows connecting digital inputs and outputs, which can be used for tracking of auxiliary states, e.g., a press of a trigger button on a tool.

### 3.2. Sensor Docking Interface

**Figure 3:**
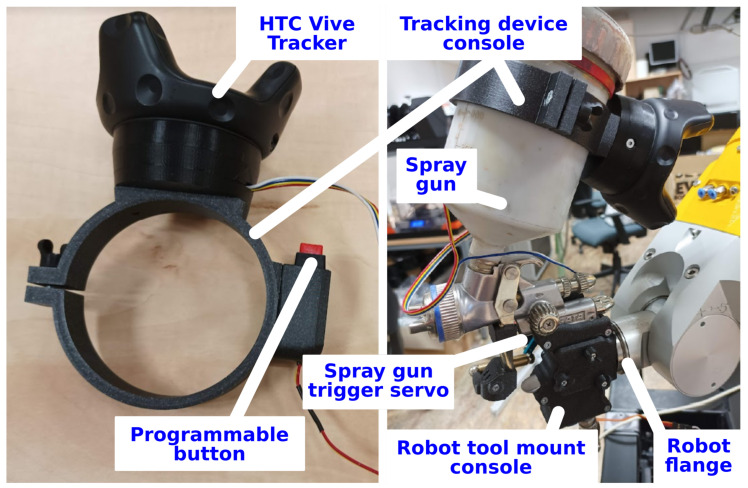
Tracking device console (**left**) with attached HTC Vive Tracker and programmable button, robot tool mount console (**right**) allowing installation of the tool to the robot flange for the automatic operation, here with the SATA jet 100 spray gun attached to a Fanuc arm.

The main part is a *tracking device console* (Figure 3 left) fulfilling the following functionalities:

- Forms a platform carrying the sensory part of the system—the HTC Vive Tracker
- Ensures attachment to a tool—spray gun was used for the robotic painting application described in this paper
- Contains a programmable button for remote control of the tracking process by the operator
- Allows sensing of auxiliary process values from the tool—e.g., activation of the spray gun in the painting scenario

The *robot tool mount console* (Figure 3 right) serves for the attachment of the working tool to the flange of a selected industrial robot.

Both consoles are to be designed for a specific tool and robotic arm to match unique requirements of a particular application [29]. They were optimised for the robotic painting cell described in this paper and manufactured through a 3D printing process. An advantage is that the same spraying hand tool used for the demonstration by the human operator can be used by the robot in the automatic mode, without a necessity of additional equipment.

### 3.3. Real-Time Data-Acquisition and Motion Control System

**Figure 4:**
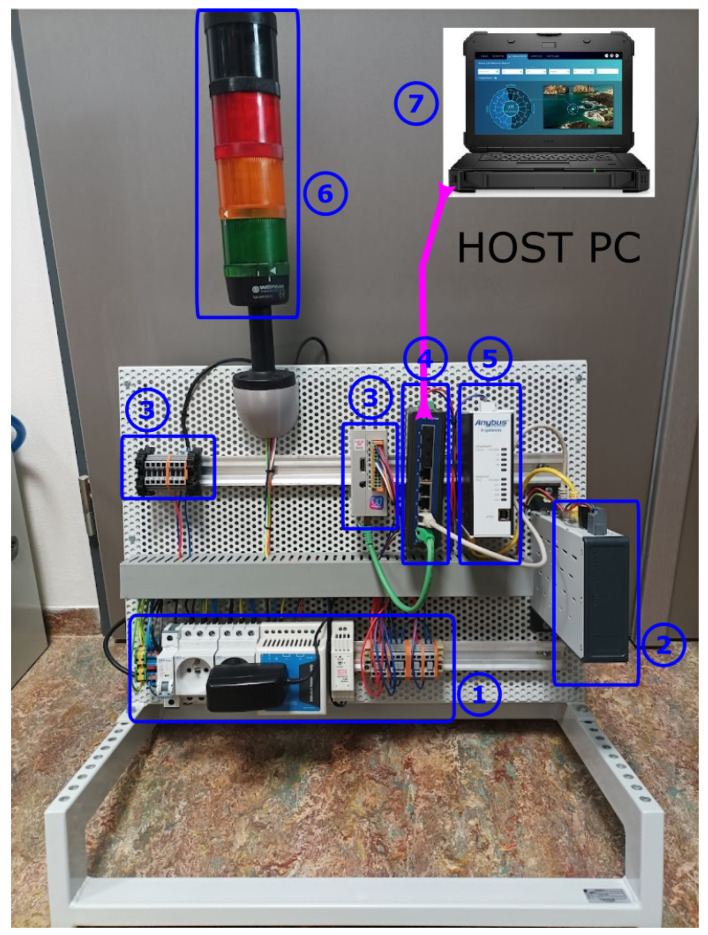
Hardware components: 1—AC/DC power supply, 2—industrial PC master controller, 3—remote I/Os, 4—Ethernet switch, 5—industrial Ethernet communication gateway, 6—state indication, 7—host computer.

A real-time controller serves as the brain of the whole system, implementing the desired functionality of capturing and reproducing demonstrated tool trajectories. The HW assembly involves the following components (Figure 4):

1. Power supply, protection and distribution —230 V AC input, 24/7.5/5 V DC output for the real-time controller, industrial Ethernet switch, EtherCAT-EthernetPI gateway, distributed I/Os and tool buttons
2. Real-time controller—B&R Automation PC 2200
3. Monarco HAT—used as remote I/Os, PWM control of tool servo control
4. Industrial Ethernet switch—Advantech EKI-2528 realising internal TCP/IP network for data communication between the individual modules
5. Anybus X-gateway—conversion between EtherCAT protocol (used at the real-time controller) and EthernetIP (standard interface to Fanuc robots, may differ for other manufacturers)
6. Industrial signal tower light—indication of tracking device state
7. Host computer—standard PC/laptop hosting a remote data post-processing application. The same device can also serve for displaying the HMI interface.

## 4. Software Components and Their Implementation

The software part of the system consists of several modules running either in the real-time controller, remote I/O device or host PC (Figure 1). Following components are employed:

- HTC Vive Tracker interface—allows low-level communication between the real-time controller and tracker system, based on *libsurvive* library [30]
- Robot controller interface—serves for communication with the robot arm. Currently available for Fanuc, Stäubli and Universal robots.
- Trajectory processing and replication module—performs data-processing of recorded motion trajectories and is also responsible for their reproduction by means of the robot arm in the automatic regime of operation
- Tracker calibration module—initialises calibration functions using a remote application, necessary for proper referencing of the motion sensor
- Motion tracking module—implements the real-time trajectory recording during the teaching phase
- Data post-processor—allows to adjust recorded motion data to fit the needs of both the user (smoothing, feed-rate adjustments) and the robotic arm (sampling period, actuator constraints)
- Human-machine interface HMI—operator screens visualising robot and tracking device state

The modules embedded in the real-time controller were implemented in REXYGEN control system [31]. Most of the application development is based on graphical programming without hand-coding with a workflow very similar to Matlab-Simulink environment. Python or C language scripting and sequential function chart support are also included, allowing the development of complex user applications. The algorithms are configured via REXYGEN Studio and compiled for real-time usage using *RexComp* tool. The application software is consequently transferred to the target platform and executed in a run-time core of the whole system *RexCore*. The SW bundle also contains HMI Designer application, which was used for the configuration of the web-based human-machine interface.

## 5. Implementation Details

Implementation details regarding essential software parts of the system follow. A complete description of the whole system is out of the scope of this paper. Only modules independent of a particular target application or robot type are described for brevity. We focused on generic functionalities transferable to similar tracking system designs, which may be beneficial for readers working on similar problems.

### 5.1. HTC Vive Tracker Interface

HTC Vive Tracker uses a closed communication protocol that connects to the SteamVR library, part of the Steam service. Using this interface in robotic applications that do not require a virtual reality goggles connection is very complicated.

Fortunately, the open-source library *libsurvive* allows client applications to connect directly to HTC Vive tracker and other peripherals for virtual reality. Libsurvive is a set of tools and libraries enabling monitoring of 6 DoF motion with the help of lighthouse base stations and moving tracker components. The library is entirely open-source and can run on any device. It currently supports both SteamVR 1.0 and SteamVR 2.0 devices and should work for all commercially available tracking systems. This library is implemented in C++ and contains a direct link to the Python language. For the needs of monitoring objects in the task of the REXYGEN control system, it was necessary to interconnect these technologies. As part of the system development, a module written in Python was created, which depends on the libsurvive library and communicates with other components using the open MAVLink protocol by means of an open-source library pymavlink. In order to be able to periodically process data from the tracker, respond to incoming messages from the MAVLink network and generate relevant information about the position and rotation of the monitored tracker, several threads must be available in the application that process data and communication at the same time. The RxPy library is used for this [32]. It is a library that supports reactive event-based programming.

The SW module is run as a service using the system daemon (systemd). As soon as the service is started, the module begins periodic communication on the configurable UDP port, listening for incoming messages in the MAVLink format and sending a HeartBeat message (HB basic message of the MAVLink protocol). Once it receives an HB message, it initializes the libsurvive library and starts periodically sending the acquired position data from the HTC Tracker.

The communication interface is mapped to standard MAVLink messages (see [33]) as follows:

- VISION_POSITION_ESTIMATE—includes x,y,z coordinates of first detected tracker position and quaternion representing the rotation
- MANUAL_CONTROL—transmits information about auxiliary tool variables, e.g., a press of a button
- COMMAND_LONG messages—allow archiving and transmission of debug logs

### 5.2. Tracking Device Calibration Module

**Figure 5:**
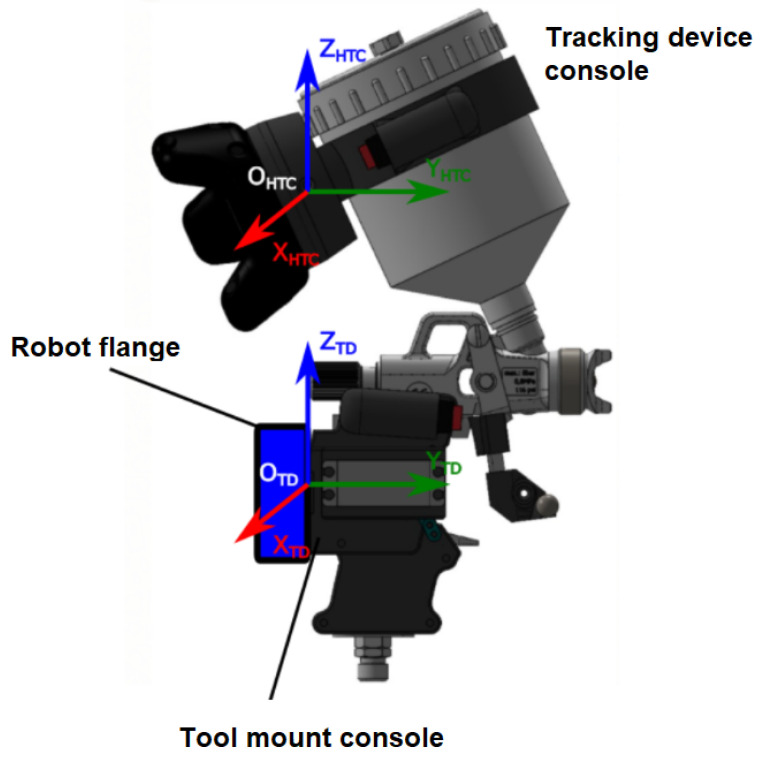
Tracking device calibration—referring tracker pose with respect to robot flange.

Calibration of the tracking device is a key task used to identify the relative position and orientation of the coordinate system (CS) of the tracking device

(1) $$F_{TD}\overset{\Delta }{=}O_{TD}-x_{TD},y_{TD},z_{TD},$$

which is aligned to the robot flange and HTC Vive Tracker coordinate system (Figure 5)

(2) $$F_{HTC}\overset{\Delta }{=}O_{HTC}-x_{HTC},y_{HTC},z_{HTC}.$$

The resulting transformation found between the $F_{TD}$ and $F_{HTC}$ coordinate frames then allows to apply robot tool path correction in order to correctly replicate the recorded and processed data from the learning process. The reason for that is that the robot motion controller only accepts trajectory data in one of the reference systems used by the robot, making the flange CS the most viable option. An alternative approach would be a definition of a new tool CS directly in the robot controller based on the calibration data. However, this would require an additional unnecessary configuration step that is difficult to achieve automatically without the assistance of the human operator, which makes the system prone to errors.

The calibration process is realised by a gradual approach to the robot arm’s defined position points, recording the tracker output, averaging the data, and calculating the resulting transformation in the data post-processor.

We assume that the kinematic structure of the robot arm to be used is known precisely, including all necessary parameters such as link lengths and relative orientation of rotation axes. Manufacturers of industrial robots commonly provide such data, or they are known in the case of custom robot design. Therefore, it is possible to compute the robot flange position and attitude from the actual measured joint positions. Standard industrial robots achieve end-effector motion repeatability in the order of ±0.01 mm. Thus, we consider robot positioning error negligible with respect to motion tracking error achievable with the optical tracker system that is about two orders of magnitude higher. This allows us to take the robot positions as reference values for the tracker calibration. Moreover, absolute positioning accuracy is not an issue since we use a difference-based method, as will be described further.

The HTC Vive tracker measures relative translation in the x−y−z Cartesian coordinates complemented by rotation encoded in the unit quaternion with respect to a CS defined during a sensor referencing procedure, which is an initialisation sequence executed as a function in the tracker firmware. The actual measured pose can be expressed by means of a homogeneous transformation matrix (HT)

(3) $$T_{tr}^{init}=\left[\begin{array}{cccc} & R_{tr}^{init} & & O_{tr}^{init}\\ 0 & 0 & 0 & 1 \end{array}\right],$$

where $R_{tr}^{init}$ is a rotation matrix (derived from the quaternion) and $O_{tr}^{init}$ is a translation vector of the tracker CS $F_{tr}$ with respect to CS $F_{init}$  (with $F_{i}$ designating the CS with origin $O_{i}$ and rectangular coordinate axes $x_{i},y_{i},z_{i}$).

The referencing is executed with the tracker attached to the robot flange. The initial pose of the robot is defined again as $T_{arm}^{init}$ (a corresponding HT matrix with the same structure as in (3)).

The robot arm moves from the initial position held during the initial tracker referencing. Any change in the end-effector pose is reflected in the change of the corresponding homogeneous transformation matrix

(4) $$T_{arm}^{init}\to T_{arm}.$$

**Figure 6:**
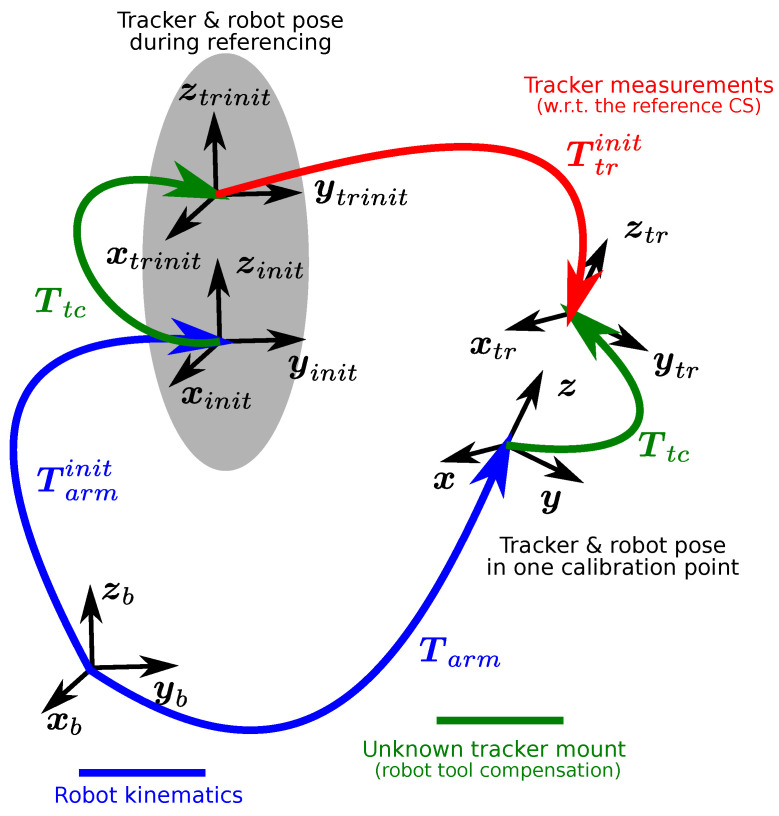
Tracking device calibration—kinematic diagram of one measurement step.

This situation is depicted in the kinematic diagram of Figure 6 showing the transition from the initial tracker initialisation phase to one measurement step of the calibration procedure.

The following coordinate systems are involved:

$T_{arm}^{init},T_{arm}$: End-effector pose of the robot during the initialisation and tracker calibration measurement phases, they are known from the robot kinematic model and measured joint positions.
$T_{tr}^{init}$: Measurements obtained from the tracker with respect to the reference CS chosen during the sensor initialisation.
$T_{tc}$: Unknown pose of the tracker with respect to robot flange, to be determined by the calibration process.

The kinematic chain shown in Figure 6 can be expressed by means of the HT matrices of the individual coordinate systems as:

(5) $$\begin{array}{c} T_{arm}^{init}\cdot T_{tc}\cdot T_{tr}^{init}=T_{arm}\cdot T_{tc}. \end{array}$$

This equality can be used for the derivation of unknown tool compensation

(6) $$T_{tc}=\left[\begin{array}{cccc} & R_{tc} & & O_{tc}\\ 0 & 0 & 0 & 1 \end{array}\right].$$

#### 5.2.1. Attitude Calibration

The Equation (5) can be rewritten in terms of the corresponding rotation matrices as:

(7) $$\begin{array}{c} R_{arm}^{init}\cdot R_{tc}\cdot R_{tr}^{init}=R_{arm}\cdot R_{tc}. \end{array}$$

By using the property of orthogonality of the rotation matrices giving $R^{T}=R^{-1}$, we can write:

(8) $$\begin{array}{cc} R_{tr}^{init} & ={\left(R_{tc}\right)}^{T}\cdot \underset{\overset{\Delta }{=}R_{arm}^{dif}}{\underset{︸}{{\left(R_{arm}^{init}\right)}^{T}\cdot R_{arm}^{}}}\cdot R_{tc}^{},\\ R_{tc}^{}\cdot R_{tr}^{init} & =R_{arm}^{dif}\cdot R_{tc}^{}, \end{array}$$

where $R_{arm}^{dif}$ denotes relative (differential) change of orientation of the robot flange CS in the measurement point with respect to its pose during the tracker initialisation, $R_{tr}^{init}$ is the attitude measured by the tracker and $R_{tc}$ is the unknown orientation of the tracker to be derived.

The Equation (8) can be rewritten as

(9) X·A=B·X,

which is the Sylvester equation with a zero right-hand side, with the known coefficient matrices $A\in R^{3,3}$, $B\in R^{3,3}$ and the matrix of unknowns $X\in R^{3,3}$ defined as:

(10) $$X\overset{\Delta }{=}R_{tc}^{},\;A\overset{\Delta }{=}R_{tr}^{init},\;B\overset{\Delta }{=}R_{arm}^{diff}.$$

The Sylvester Equation (9) can be transformed to an equivalent set of linear equations

(11) $$\underset{\in R^{9,9}}{\underset{︸}{\left(I_{3\times 3}⊗B-A^{T}⊗I_{3\times 3}\right)}}\cdot \underset{\in R^{9,1}}{\underset{︸}{\mathrm{vect}X}}=O,$$

where ⊗ denotes the Kronecker matrix product, $I_{3\times 3}$ is the identity matrix and the unknown matrix X is reshaped into the vector

(12) $$\mathrm{vect}X=\left[\begin{array}{c} X(:,1)\\ X(:,2)\\ X(:,3) \end{array}\right],$$

 with X(:,i) denoting a selection of an *i*-th column of the matrix X.

For *N* different calibration points approached by the robot, we get

(13) M·vectX=0,

where

$$M=\left[\begin{array}{c} m_{1}\\ m_{2}\\ \vdots \\ m_{N} \end{array}\right]\in R^{9N,9},\;m_{i}=\left(I_{3\times 3}⊗B_{i}-A_{i}^{T}⊗I_{3\times 3}\right)\;(\mathrm{for}\;\mathrm{the}\;i-\mathrm{th}\;\mathrm{point}).$$

This can be further rearranged to the form of:

(14) $$\underset{\in R^{9,9}}{\underset{︸}{\left(M^{T}\cdot M\right)}}\cdot \mathrm{vect}X=0.$$

The matrix $\left(M^{T}\cdot M\right)$ in the homogenous Equation (14) is singular from the principle of its construction. Therefore, there always exists a nontrivial solution vectX≠0. However, the consistence of the equation is lost in practice due to the real data that are corrupted by various measurement errors. However, suitable solution can still be found by formulating the following optimisation problem

(15) $$\mathrm{vect}X^{🟉}=\min_{\mathrm{vect}X}\frac{∥\left(M^{T}\cdot M\right)\cdot \mathrm{vect}X∥}{∥\mathrm{vect}X∥},$$

where ∥🟉∥ is the $L^{2}$ vector norm.

The problem (15) is essentially a least-squares minimisation of residuals in the linear equation system (13) leading to the search for a direction with the smallest $L^{2}$ gain of the $M^{T}\cdot M$ matrix operator. From the properties of singular values of a matrix, the solution exists in the form of

(16) $$\mathrm{vect}X^{🟉}=v_{\min}^{R}\left(M^{T}\cdot M\right),$$

where $v_{\min}^{R}$ is the right singular vector corresponding to the smallest singular value $\underline{\sigma }\left(M^{T}\cdot M\right)$.

From the back-substitution done in (10), we get the rotation matrix $R_{\mathrm{tc}}=X^{*}$, with the columns assembled from (16). Gramm-Schmidt process is used as a last step to correct potential loss of orthogonality due to numerical errors.

We may note that a solution cX with c∈R being an arbitrary scalar is also the solution of (9). This ambiguity is removed by determining a correct sign of *X* from the assumed condition of right-handed CS, i.e., $\det(R_{tc})=1$. The absolute value of *c* does not change the physical meaning of the CS axes and can be scaled to achieve unit vector lengths in the columns of $R_{\mathrm{tc}}$.

Error of the estimated model can be obtained from the acquired optimal solution in (16) by computing the Standard error of the regression as:

(17) $${s^{2}}_{rot}=\frac{1}{N-3}\cdot {∥M\cdot \mathrm{vect}X^{*}∥}^{2},$$

where N−3 are statistical degrees of freedom in the orientation estimate problem.

#### 5.2.2. Translation Calibration

The Equation (5) describing the kinematic chain loop can be arranged analogously to the case with rotations in (7) as follows:

(18) $$T_{tc}^{}\cdot T_{tr}^{init}=T_{arm}^{dif}\cdot T_{tc}^{},\;\left[\begin{array}{cccc} & R_{tc}\cdot R_{tr}^{init} & & O_{tc}+R_{tc}\cdot O_{tr}^{init}\\ 0 & 0 & 0 & 1 \end{array}\right]=\left[\begin{array}{cccc} & R_{arm}^{diff}\cdot R_{tc} & & O_{arm}^{diff}+R_{arm}^{diff}\cdot O_{tc}\\ 0 & 0 & 0 & 1 \end{array}\right],\;\left(I_{3\times 3}-R_{arm}^{diff}\right)\cdot O_{tc}=O_{arm}^{diff}-R_{tc}\cdot O_{tr}^{init},$$

where $R_{tc}$ is rotation of tool compensation known from the previous step and $O_{tc}$ is the translation vector to be found.

Equation (18) defined for a single measurement point can again be extended to generic case of *N* different calibration positions:

(19) $$\left[\begin{array}{c} I_{3\times 3}{-}^{1}\;R_{arm}^{diff}\\ \vdots \\ I_{3\times 3}{-}^{N}\;R_{arm}^{diff} \end{array}\right]\cdot O_{tc}=\left[\begin{array}{c} {}^{1}\;O_{arm}^{diff}-R_{tc}\cdot^{1}\;O_{tr}^{init}\\ \vdots \\ {}^{N}\;O_{arm}^{diff}-R_{tc}\cdot^{N}\;O_{tr}^{init} \end{array}\right]$$

where ${}^{i}\;O_{arm}^{diff}$, ${}^{i}\;R_{arm}^{diff}$ and ${}^{i}\;O_{tr}^{init}$ corresponds to an *i*-th measurement.

The overdetermined set of linear equations (19) does not have an exact solution. However, optimal approximation in the least squares sense can be derived as follows:

(20) $$O_{tc}^{*}={\left(K_{1}^{T}\cdot K_{1}\right)}^{-1}\cdot K_{1}^{T}\cdot K_{2},$$

where

$$K_{1}\overset{\Delta }{=}\left[\begin{array}{c} I_{3\times 3}{-}^{1}\;R_{arm}^{diff}\\ \vdots \\ I_{3\times 3}{-}^{N}\;R_{arm}^{diff} \end{array}\right],K_{2}=\left[\begin{array}{c} {}^{1}\;O_{arm}^{diff}-R_{tc}\cdot^{1}\;O_{tr}^{init}\\ \vdots \\ {}^{N}\;O_{arm}^{diff}-R_{tc}\cdot^{N}\;O_{tr}^{init}. \end{array}\right]$$

The Moore-Penrose inverse in (20) can be computed in a numerically robust way using Singular-value decomposition [34]. Error of the fit can again be evaluated as:

(21) $${s^{2}}_{trans}=\frac{1}{N-3}\cdot {∥K_{1}\,O_{tc}^{*}-K_{2}∥}^{2}.$$

The error of the tool compensation calibration process contributes to the overall tracking error of the sensory system. This precision was experimentally validated using the HTC Vive sensor and industrial robot arm. The results are shown in Section 6.

### 5.3. Data Post-Processing SW Module

This part of the tracking system software is responsible for proper adjustment of the recorded motion data prior to its automatic replication by the robotic arm. The post-processor module is implemented as a Matlab application that continuously runs on a host PC and monitors commands sent by the real-time controller using RESTful API [35]. Large data files transferred via CSV files using FTP protocol.

The raw pose data are acquired and sampled from the HTC Vive tracker with a period of 10 ms. They are transformed into the robot tool coordinate system using the tracker referencing and calibration system described in the Section 5.2. Next, various data processing steps follow:

1. Zero-phase low-pass filtering—this step is needed to remove measurement noise and operator-induced vibrations. Translation and attitude data are handled separately.
2. Robot inverse kinematics computation—the recorded tool trajectory is transformed to joint coordinates of the used robotic arm using a known kinematic transform. This step has to resolve ambiguity in robot configuration, as there are eight joint space solutions corresponding to the same tool pose for a typical 6DoF industrial robot. The user can select from a particular solution, generate all the available solutions or switch to a particular solution corresponding to the actual state of the robot. The last option is viable for situations where the operator may be able to manually position the robot arm to a configuration suitable for the intended task.
3. Feedrate adjustment—the pose of the tool can be reproduced by the robot exactly in the time domain as recorded by the operator. Another option is to preserve only the geometric shape and adjust the feed rate (tangential velocity) of the motion performed by the robot. The user can choose between these two interpolation modes and change the feed rate in the latter as needed by the application.
4. Singular positions check—reproducibility of the recorded motion by the particular robot in use is checked with respect to possible crossing of singular positions, either inside or at the border of the robot workspace. The occurrence of such events is undesirable and has to be resolved, either by adjusting the desired trajectory, repositioning the robot base in its workspace, or choosing different joint configurations from the permissible set given by the inverse kinematics.
5. Robot drives limits check—reproducibility of the motion at the drive level is evaluated by detecting possible violations of maximum position, velocity, acceleration and optionally jerk limits for the individual robot axes. Position overruns can be removed similarly to the occurrence of singular positions. The magnitude of the higher derivatives of position is dominantly determined by the feed rate, that can be reduced when needed. The proximity of singular points also often leads to rapid joint motions. Therefore, cures for singular point resolution usually improve the physical feasibility at the drive level as well.
6. Resampling or interpolation—the resulting motion profiles, now readily available in the joint coordinates, are resampled to a different update rate suitable for the particular robot arm and its controller. For this sake, a shape preserving cubic spline interpolation implemented in Matlab’s interp1 function was used [36].

#### 5.3.1. Trajectory Smoothing via Zero-Phase Filtering

Processing the raw data from the tracker sensor is a fundamental step necessary to produce smooth motions reproducible by the robot arm. Low-pass filtering helps mitigate sensor noise and unwanted vibrations induced by the operator’s hand wielding the tool during the teaching phase. This step is performed offline, and the availability of the whole data record is used to employ non-causal zero-phase filtering. This allows modification of the amplitude spectrum of the motion data in the high-frequency range without introducing a significant phase delay that would distort the shape of the originally recorded trajectory.

There are two basic approaches to zero-phase filtering commonly used. First technique is the *forward-backward filtering* algorithm using IIR filters, implemented e.g., in the filtfilt function of Matlab [37]. The basic idea behind this approach is to utilise the time-reversal property of the Z-transform and Discrete Fourier transform.

For a real discrete-time sequence x[n], there exists a Z-transform image defined as the formal power series

(22) $$Z\left(x\left[n\right]\right)=X\left(z\right)\overset{\Delta }{=}\underset{n=-\infty }{\sum^{\infty }}x\left[n\right]z^{-n},$$

where *z* is a complex number.

For a time-reversed sequence x[−n], the corresponding image is obtained as

(23) $$Z\left(x\left[-n\right]\right)=\underset{n=-\infty }{\sum^{\infty }}x\left[-n\right]z^{-n}=\underset{m=-\infty }{\sum^{\infty }}x\left[m\right]z^{m}=\underset{m=-\infty }{\sum^{\infty }}x\left[m\right]{\left(z^{-1}\right)}^{-m}=X\left(z^{-1}\right).$$

The forward-backward filtering algorithm uses an LTI filter with a transfer function

(24) $$H\left(z\right)=\frac{Y(z)}{U(z)},$$

where Y(z),U(z) denote Z-transform images of output and input sequences of the filter.

The first step of the algorithm employs the smoothing filter to process the input data

(25) Y(z)=H(z)U(z).

The resulting output is then time-reversed, which corresponds to the image

(26) $$T_{r}\left(z\right)=Y\left(z^{-1}\right)=U\left(z^{-1}\right)H\left(z^{-1}\right).$$

The filter *H* is applied again to produce a new output

(27) $$Z\left(z\right)=H\left(z\right)T_{r}\left(z\right)=U\left(z^{-1}\right)H\left(z^{-1}\right)H\left(z\right).$$

Second time-reversal of *Z* follows, giving the final filtered sequence with the image

(28) $$F\left(z\right)=Z\left(z^{-1}\right)=U\left(z\right)H\left(z^{-1}\right)H\left(z\right)\overset{\Delta }{=}U\left(z\right)H_{zp}\left(z\right),$$

where $H_{zp}$ is a total transfer function of the whole filtering procedure.

Frequency response function can be obtained from (28) by substituting $z=e^{i\omega }$

(29) $$F\left(e^{i\omega }\right)=U\left(e^{i\omega }\right){\left|H\left(e^{i\omega }\right)\right|}^{2},$$

revealing that the double filtered signal spectrum is adjusted in amplitude, but zero-phase distortion is achieved. Practical implementation of the algorithm requires a careful choice of initial conditions to avoid excessive transients at the edges of the filtered signal [38].

A second common approach is a synthesis of a linear-phase FIR filter and its anti-causal application to the filtered signal.

The transfer function of the FIR filter is assumed in the form of

(30) $$H\left(z\right)=b_{0}+b_{1}\left(z+z^{-1}\right)+b_{2}\left(z^{2}+z^{-2}\right)+\ldots +b_{n}\left(z^{n}+z^{-n}\right)=b_{0}+\underset{n=1}{\sum^{n}}b_{n}\left(z^{n}+z^{-n}\right),$$

which corresponds to a finite impulse function sequence of length 2n+1 with a symmetrical distribution of n+1 coefficients available as design parameters

(31) $$h\left(k-n\right):=\left\{b_{n},b_{n-1},\ldots ,b_{2},b_{1},b_{0},b_{1},b_{2},\ldots ,b_{n-1},b_{n}\right\}.$$

The filter is anti-causal and requires a preview time of *n* samples to determine its current output.

The zero-phase property of such filter is revealed by computing its frequency response function

(32) $$H\left(e^{i\omega }\right)=b_{0}+\underset{n=1}{\sum^{n}}b_{n}\left(e^{i\omega n}+e^{-i\omega n}\right)=b_{0}+\underset{n=1}{\sum^{n}}2b_{n}\cos\left(\omega n\right),$$

which shows that the phase response of the filter is real, thus it can be either 0 or π radians. As long as the coefficients $b_{i}$ are properly chosen such that there are no abrupt changes in phase in the filter passband, it can be used for zero-phase filtering.

There are many methods for the derivation of proper IIR or FIR low-pass filters, see e.g., ref. [39]. The choice of the filter dynamics proved to be crucial for its successful application to motion data post-processing. Otherwise, an issue of unwanted motion oscillations may appear. This problem is discussed in Section 6 devoted to experimental results.

#### 5.3.2. Robot Motion Simulation Using Virtual Model

The trajectories for a particular robot type prepared by the post-processor module can be simulated in a virtual environment to validate that the motion task was learned correctly.

**Figure 7:**
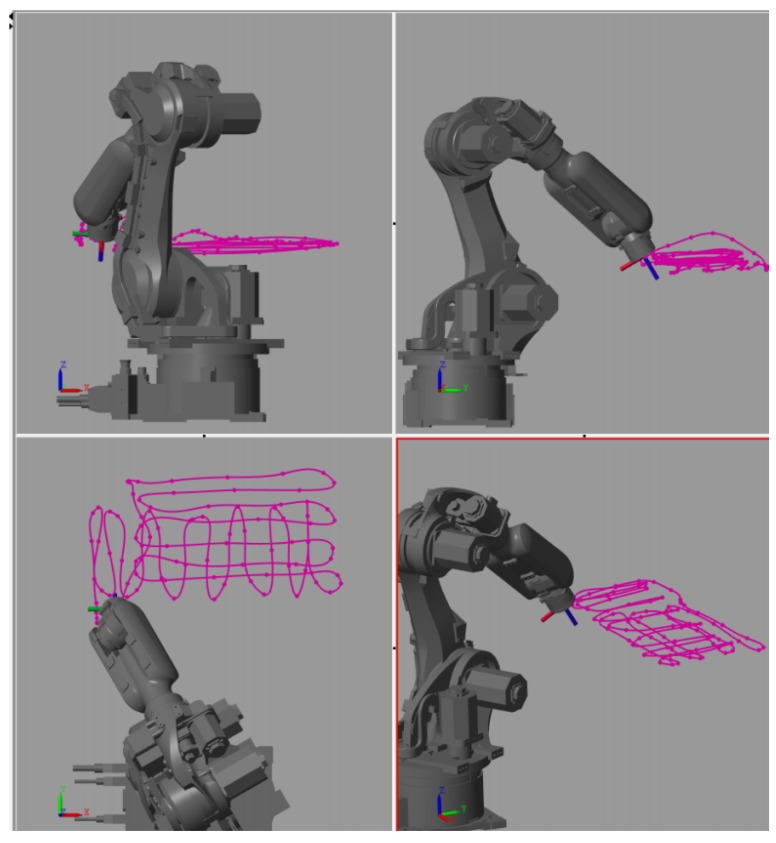
Robot motion simulation module—3D visualisation showing the resulting trajectory of the robot, allowing a final inspection of the computed motion trajectories.

Digital Twin of the robot is implemented in the form of the direct kinematic model that receives the precomputed motion data in the form of joint coordinates. The model simulates the resulting motion of the robot arm (Figure 7). Collisions of the robot with obstacles in its workspace or collisions between the individual robot links can be revealed here.

After this final check, the motion trajectories are uploaded back to the real-time controller that subsequently commands the controller of the robot and reproduces the learned motions on demand.

### 5.4. Human-Machine Interface

A human-machine interface is implemented in the real-time controller. It can be accessed via a running web server using any device equipped with a standard web browser supporting HTML5, i.e., desktop PC, laptop, mobile phone or tablet.

**Figure 8:**
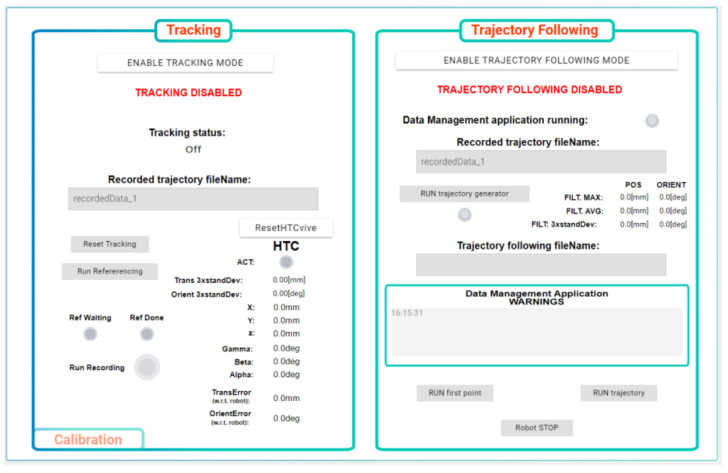
Human machine interface—high-level part for tracking system and trajectory following.

**Figure 9:**
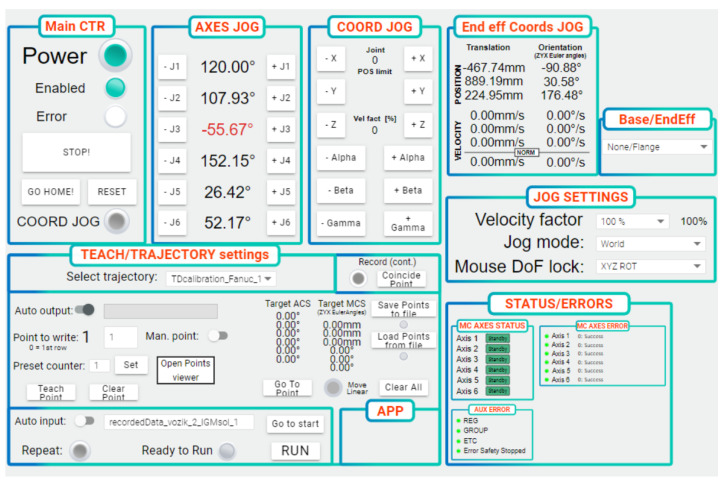
Human machine interface—low-level part for robot motion control.

The HMI application comprises several screens allowing to access individual functions of the robotic cell equipped with the tracking system. The high-level part shown in Figure 8 deals with the functions associated with the tracking device and trajectory following. The user can calibrate the tracker and activate the recording of the new motion trajectory demonstrated by the operator. Once the processed motion data for the robot are available from the post-processor, they can be uploaded to the robot. The low-level part of the HMI serves for direct control of the robot in use (Figure 9). The robot can be switched on or off, automatically repositioned to a defined home configuration, or operated in a manual jog regime, either in an uncoordinated manner by means of individual joints or using a coordinated jog in a chosen machine coordinate system. Errors reported by the robot motion controller are shown for the purpose of diagnostics and system commissioning.

## 6. Experimental Results

### 6.1. Evaluation of Pose Estimation Error

The tracking performance of the whole system was evaluated through a Fanuc LRMate 200iD robotic arm that served as a reference measurement device providing ground-truth data. The measurement procedure was performed by executing the following sequence of operations:

1. Attachment of the HTC Tracker to a tool fixed to the robot flange
2. Sensor initialisation and execution of the calibration sequence, as described above
3. Adjustment of robot configuration to follow several measurements positions while recording the pose data of the flange reported by the robot and relative pose data measured by the tracker
4. Converting the tracker data to the pose of the robot flange
5. Comparison of translation and orientation error between the two coordinate systems reported by the robot (reference ground-truth value) and tracker

The overall pose estimation error comes from three main sources:

- Calibration error of the tracker coordinate system—results from the errors in the relative pose of the tracker sensor with respect to the robot flange. This coordinate transform is estimated during the calibration procedure described in Section 5.2. This error is directly propagated to the computed motion trajectories for the robot, resulting in a systematic bias in the translation and orientation of the tool during the automatic motion reproduction. The error is deterministic by nature. Its magnitude can be reduced by proper execution of the calibration procedure, in particular by covering the relevant workspace with a sufficient amount of calibration points and averaging multiple measurements to reduce the variance of the estimates.
- Tracking sensor error—results from various sources of internal errors of the tracking device, partly due to measurement noise in the optical flow, signal losses between the tracker and base stations and motion induced errors affecting mainly the IMU part of the system. This error is a mix of deterministic and stochastic components and can hardly be reduced without altering the signal processing routines implemented in the sensor firmware.
- Post-processing error—application of the smoothing filter to the raw recorded motion data alters the original trajectory and potentially introduces a systematic error. Although using a zero-phase algorithm, improper choice of filter bandwidth may still lead to severe performance deterioration. This source of error can be mitigated by analysing the spectral content of the raw data, which usually reveals a frequency range relevant for motion tracking, allowing it to be separated from the measurement noise by filtering.

**Figure 10:**
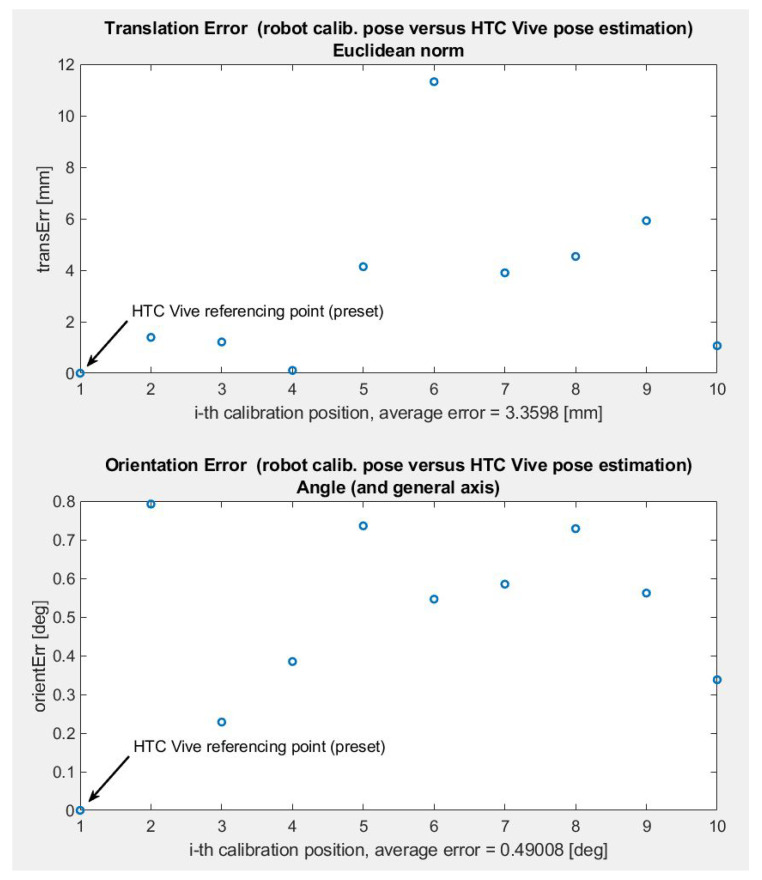
Tracking error evaluation—static pose estimation test using discrete measurements.

Figure 10 shows the results of one such error evaluation sequence for a specific choice of ten measurement points. This test focused on static accuracy, emphasising the systematic bias introduced by calibration and tracker sensing errors. The first point corresponds to a referencing point of the HTC Tracker. Therefore, the pose estimation error is zero. Once the robot transitions to nine other evaluation positions, translation error is evaluated using the Euclidean distance between actual and reported CS origins. The orientation error is computed as the value of relative rotation around a generic axis between the reference and measured CSs. Average translation error is in the order of lower units of millimetres, while the orientation error is about a half of a degree.

**Figure 11:**
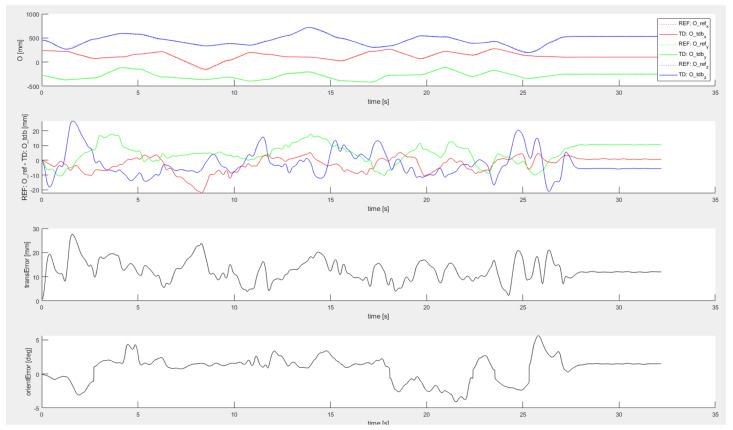
Tracking error evaluation—dynamic pose estimation test using continuous tracking, from top to bottom—reference and estimated flange position, position error in three principal directions, Euclidean translation error, orientation error.

Another series of experiments was conducted to evaluate the influence of tracked motion dynamics. Instead of a discrete set of evaluation points, continuous trajectories of various shapes and different velocity/acceleration profiles were tracked and compared with the reference data from the robot arm. Example of such dynamic test is shown in Figure 11. As can be seen from the plots, the worst-case error in this dynamic experiment is about 5-times higher than during the static case. By comparing the CS translation data with the error evolution plot, it can be seen that the accuracy was worst during rapid translation motions. Similar performance was achieved during tracking of other motion trajectories.

The overall accuracy achieved during field tests with 10 different carefully designed motion profiles are as follows:

- Maximum achieved translation error [mm]: 44.91
- Mean value of achieved translation error [mm]: 8.82
- Maximum achieved orientation error [deg]: 8.17
- Mean value of achieved orientation error [deg]: 1.58

A probable explanation for the observed performance degradation during highly dynamic motions comes from the influence of the IMU employed in the tracker sensor. This is due to the embedded accelerometers that are affected by rapid translation movements from the principle of operation; the gravity vector sensed in the steady state gets deflected, affecting precision of attitude estimates. Since the HTC Tracker is primarily intended for gaming industry, the IMU presumably receives a higher relative weight with respect to the optical sensor in the internal sensor fusion process, prioritising rapid and smooth response over absolute tracking error magnitude.

The achieved results are consistent with the tests of the stand-alone HTC tracker reported in the literature [27,28]. This leads us to the conclusion that the tracking sensor error is a dominant factor determining the overall performance and the calibration and post-processing algorithms were designed correctly. The achieved pose estimation accuracy meets the formulated design requirements, making the system viable for intended motion control applications.

Detailed parameters and shape of the motion profiles used during the dynamic tracking experiments are shown in the Appendix A in Table A1.

### 6.2. Zero-Phase Filter Design for Motion Post-Processing

Both the zero-phase filtering algorithms presented in Section 5.3.1 require a careful design of the IIR or FIR low-pass filter. Apart from the choice of filter bandwidth, another fundamental issue was revealed during the development of the tracking system.

Many commonly used design methods introduce complex poles in the resulting filter transfer function, e.g., well-known Butterworth, Chebyshev or Elliptic filters. The complex poles are generally favourable as they allow a steeper roll-off in the transition band of filter amplitude response. However, they also introduce oscillatory impulse response that may lead to unacceptable transients in some applications. A similar effect is observed in the case of FIR filters that contain only zeros, but their impulse function contains negative coefficients. This proved to be the case for the post-processing of motion trajectory data.

**Figure 12:**
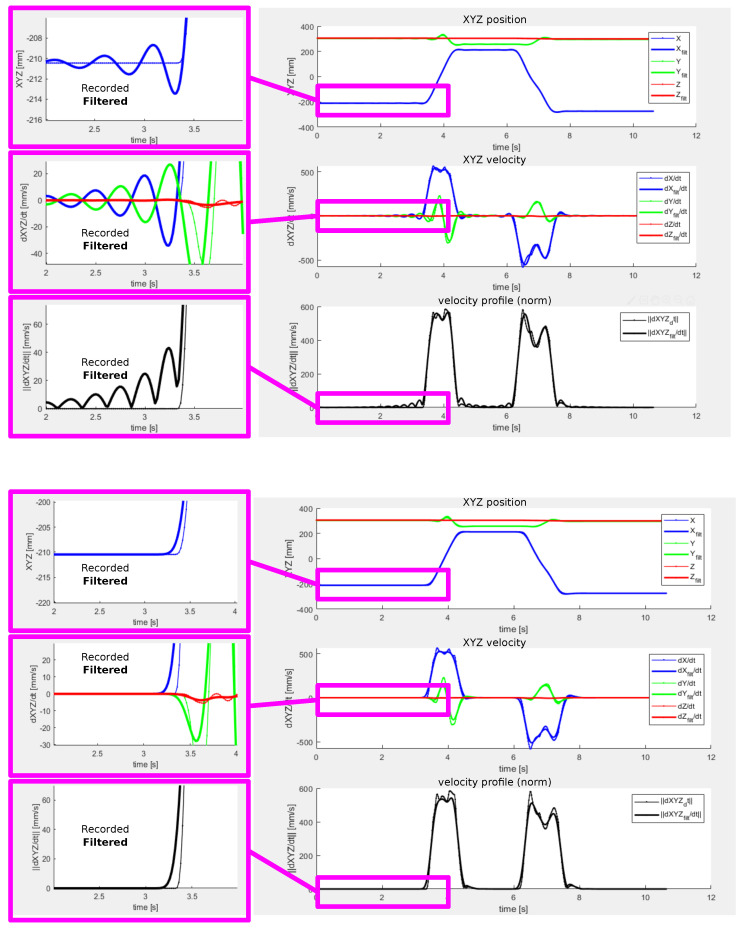
Smoothing filter induced oscillations during rapid steady-state to motion transitions—(**top**) forward-backward filtering using 6th order Butterworth filter, (**bottom**) zero-phase FIR filter with monotonous step response designed by windowing approach.

The problem introduced by oscillatory dynamics of the smoothing filter is demonstrated by Figure 12 showing the filtering step applied to a sequence of two point-to-point motions in the 3D space. The top plot corresponds to the forward-backward filtering algorithm performed with a 6th order Butterworth type low-pass digital filter. The smoothing works as expected in most parts of the motion trajectory, reducing rapid changes in the filtered signal without introducing a phase delay. However, significant oscillations are present in the sections corresponding to transitions from rest to motion and vice-versa. This effect is observed even at the beginning of the motion due to the anti-causality of the filter. The reason for this behaviour is in the oscillatory impulse function of the filter that is embedded in the transients with rapid changes in position. The resulting trajectory cannot be used as a reference for the robot since it exhibits the unwanted oscillations deviating significantly from the motion recorded during the teaching phase.

There are generally two cures for this problem:

1. Design an IIR low-pass filter for the forward-backward filtering algorithm with real poles to achieve a monotonous step response
2. Design a zero-phase FIR low-pass filter with non-negative values of impulse response, leading again to a monotonous step response

Both approaches are viable and lead to similar results. In our actual application, we opted for the latter method, focusing on FIR filters designed by the windowing approach [39]. The basic idea is to approximate the impulse function of an ideal Sinc filter that leads to “brick-wall” shape of the resulting amplitude frequency response by a suitable finite-length taper window. Various windowing functions are known from the literature, differing in the shape of the resulting filter frequency response. A compromise is sought in terms of high-frequency roll-off, transition-band shape and level of the stop-band attenuation. We opted for the Blackman-Harris symmetric window [40], which can be generated using a closed-form formula

(33) $$w\left(k\right)=a_{0}-a_{1}\cos\left(\frac{2\pi k}{N-1}\right)+a_{2}\cos\left(\frac{4\pi n}{N-1}\right)-a_{3}\cos\left(\frac{6\pi n}{N-1}\right);\;k=0,\ldots ,N-1,$$

where the $a_{i}$ are fixed scalar constants defined as

(34) $$a_{0}=0.35875,\;a_{1}=0.48829,\;a_{2}=0.14128,\;a_{3}=0.01168,$$

and *N* is an odd number defining window length that determines the resulting filter bandwidth.

The smoothing filter impulse function (30) is obtained from the designed window (33) by applying a scaling factor

(35) $$h\left(k\right)=\frac{w(k)}{\sum_{\forall k}w\left(k\right)};\;k=0,\ldots ,N-1,$$

to achieve unitary static gain.

**Figure 13:**
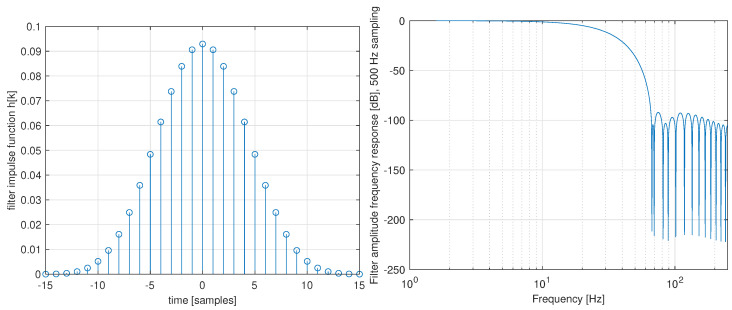
Example of Blackman-Harris zero-phase FIR filter design used motion smoothing—(**left**) filter impulse response, (**right**) amplitude frequency response.

An example of a Blackman-Harris window zero-phase FIR filter of length N=31 that was used in the experiments is shown in Figure 13. The favourable shape of filter frequency response is achieved while maintaining positive values of impulse function coefficients, which was the main requirement for achieving smooth motion transients. The influence of smoothing filter dynamics on the robot motion response is documented in a movie shared via a hyperlink given in Appendix B.

### 6.3. Pilot Application—Robotic-Assisted Spray Painting

**Figure 14:**
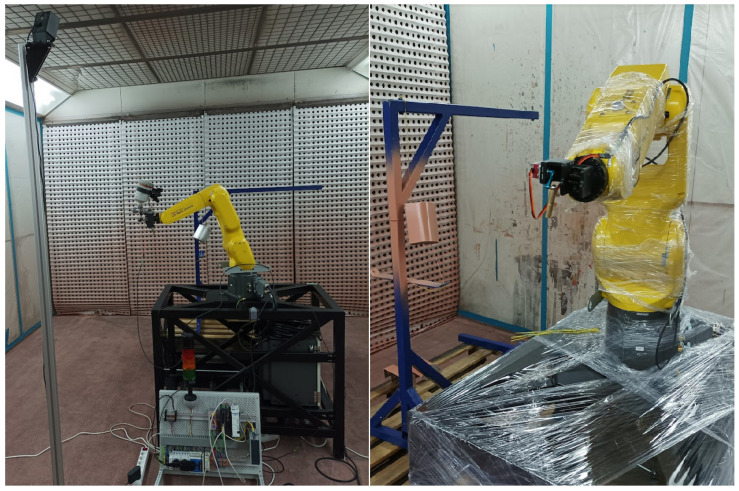
Pilot application—flexible spray coating robotic cell, (**left**) Fanuc LRMate 200iD robotic arm equipped with the tracking system, (**right**) detailed view.

The developed tracking system was employed in a pilot operation in a robotic work-cell intended for automated small series production of painted parts. Fanuc LRMate 200iD robotic arm was equipped with the motion tracker and the whole system was installed in a paint shop at the workplace of Lasertherm company, which is our industrial partner dealing with robotic automation in various domains. Figure 14 shows the robot arm with an attached paint gun and HTC Vive sensor. Tracker base station attached to an adjustable pillar is visible on the left.

In the teaching phase, an operator experienced in spray coating activates the tracking system and demonstrates the intended task. The recorded and post-processed trajectory is further used for automatic replication of the task by the robot. The goal is to automate the painting process using the robot for small to medium series of jobs, where traditional methods of robot programming fail to be cost and time-effective.

**Figure 15:**
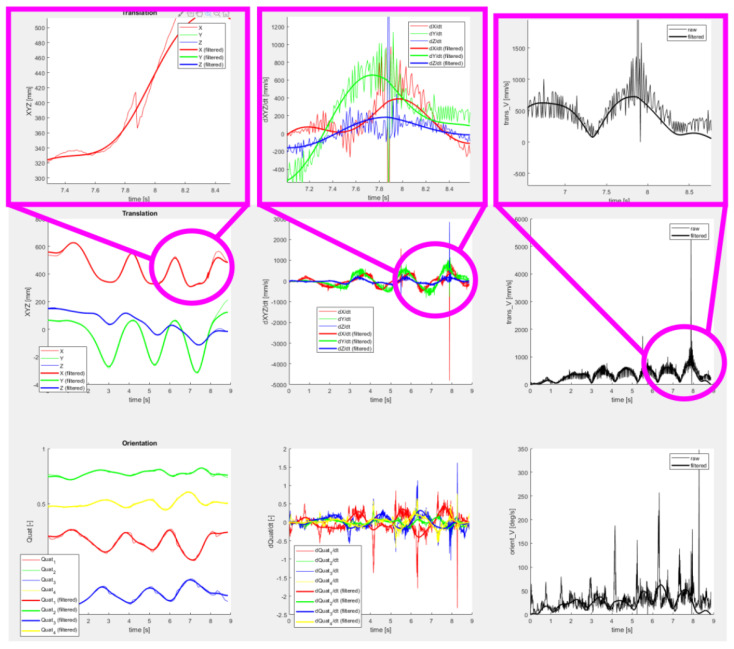
Pilot application—example of spraying motion task recorded by operator and post-processed by the software, (**top**) details of specific parts of the trajectory showing the smoothing functionality of the post-processor, (**center**) translation data in the individual axes showing position, velocities and feedrate, (**bottom**) orientation data showing quaternion elements, their derivatives and rotational velocity.

Figure 15 shows details of a motion trajectory recorded by the operator during a spray painting task. The role of the post-processor in terms of motion smoothing is clearly demonstrated by comparing the raw data acquired from the tracking sensor with the filtered trajectory. The filtering step is necessary to mitigate unwanted oscillations due to measurement noise and operator’s hand tremble. The resulting motion is smooth and physically feasible by the robot arm.

A movie demonstrating the system’s functionality in the spray coating application was created. A hyperlink is provided in the Appendix B.

## 7. Discussion

The developed motion tracking system was successfully employed in a robotic cell installed in a paint shop. It proved to be usefull for flexible automation tasks requiring frequent reconfiguration of robot motion trajectories. The utilisation of the affordable HTC sensor makes it attractive for the cost-effective integration of robot manipulators in applications with moderate tracking precision requirements. Programming of the motion trajectories is intuitive and does not require a deep background in industrial automation or robotics from the operating personnel, which reduces commissioning and running costs.

The main limitation of the system comes from the pose estimation accuracy that is inherently given by the employed motion sensor. However, the modularity of the developed system allows relatively simple replacement of the tracking sensor in case of stricter performance requirements. We are currently working on a modified version that uses a cable system with direct length measurement attached to the tool during motion recording. Preliminary results show that the tracking accuracy can be substantially improved at the cost of the introduction of minor movement restrictions for the operator. This will be addressed in future research. Another possible research direction involves modifications of the data fusion process performed directly in the tracking sensor software, aiming at the improvement of the absolute tracking accuracy.

The developed system is now ready for industrial deployment. Please contact the authors if you are interested in using the presented results in your application or research.

## Data Availability

Supporting data available at links given in the Appendix A and Appendix B.

## Abbreviations

The following abbreviations are used in this manuscript:

MDPI	Multidisciplinary Digital Publishing Institute
LED	Light-emitting diode
IMU	Inertial measurement unit
AC/DC	Alternating/dirrect cuurent
PWM	Pulse-width modeulation
HMI	Human-machine interface
I/O	Input/output
PC	Personal computer
DoF	Degree of freedom
CS	Coordinate system
HT	Homogenous transformation matrix
IIR	Infinite impulse response
FIR	Finite impulse response
HTML	Hypertext markup language
USB	Universal serial bus
REST	REpresentational State Transfer
CSV	Comma separated values
FTP	File transfer protocol

## Appendix A. Results of Motion Tracker Performance Experiments

**Table A1:** Parameters of the executed motion profiles during dynamic testing.

Testing Motion	Resulting Parameters & Graphs
TEST_1_1 (Straight line crossings without stopping) maxVelocity_translation [mm/s]: 200 maxAcceleration_translation [mm/s${}^{2}$]: 1000 maxVelocity_rotation [deg/s]: 80 maxAcceleration_rotation [deg/s${}^{2}$]: 360	Max. translation error [mm]: 27.58 Avg. translation error [mm]: 12.65 Max. orientation error [deg]: 5.63 Avg. orientation error [deg]: 1.80 Link to plots, accessed 9 June 2022
TEST_1_2 (Straight line crossings without stopping) maxVelocity_translation [mm/s]: 50 maxAcceleration_translation [mm/s${}^{2}$]: 1000 maxVelocity_rotation [deg/s]: 80 maxAcceleration_rotation [deg/s${}^{2}$]: 360	Max. translation error [mm]: 50.54 Avg. translation error [mm]: 9.41 Max. orientation error [deg]: 3.58 Avg. orientation error [deg]: 1.28 Link to plots, accessed 9 June 2022
TEST_1_3 (Straight line crossings without stopping) maxVelocity_translation [mm/s]: 400 maxAcceleration_translation [mm/s${}^{2}$]: 1500 maxVelocity_rotation [deg/s]: 80 maxAcceleration_rotation [deg/s${}^{2}$]: 360	Max. translation error [mm]: 40.16 Avg. translation error [mm]: 14.07 Max. orientation error [deg]: 12.99 Avg. orientation error [deg]: 2.75 Link to plots, accessed 9 June 2022
TEST_1_4 (Straight line crossings without stopping) maxVelocity_translation [mm/s]: 400 maxAcceleration_translation [mm/s${}^{2}$]: 1500 maxVelocity_rotation [deg/s]: 80 maxAcceleration_rotation [deg/s${}^{2}$]: 360	Max. translation error [mm]: 40.47 Avg. translation error [mm]: 14.04 Max. orientation error [deg]: 13.07 Avg. orientation error [deg]: 2.75 Link to plots, accessed 9 June 2022
TEST_1_5 (Straight line crossings with stopping) maxVelocity_translation [mm/s]: 200 maxAcceleration_translation [mm/s${}^{2}$]: 1000 maxVelocity_rotation [deg/s]: 80 maxAcceleration_rotation [deg/s${}^{2}$]: 360	Max. translation error [mm]: 43.92 Avg. translation error [mm]: 3.96 Max. orientation error [deg]: 3.85 Avg. orientation error [deg]: 1.25 Link to plots, accessed 9 June 2022
TEST_2_1 (Straight line crossings with stopping) maxVelocity_translation [mm/s]: 200 maxAcceleration_translation [mm/s${}^{2}$]: 500 maxVelocity_rotation [deg/s]: 80 maxAcceleration_rotation [deg/s${}^{2}$]: 360	Max. translation error [mm]: 50.67 Avg. translation error [mm]: 11.50 Max. orientation error [deg]: 8.81 Avg. orientation error [deg]: 1.14 Link to plots, accessed 9 June 2022
TEST_2_2 (Straight line crossings with stopping, re-calibration) maxVelocity_translation [mm/s]: 200 maxAcceleration_translation [mm/s${}^{2}$]: 500 maxVelocity_rotation [deg/s]: 80 maxAcceleration_rotation [deg/s${}^{2}$]: 360	Max. translation error [mm]: 24.82 Avg. translation error [mm]: 7.64 Max. orientation error [deg]: 6.53 Avg. orientation error [deg]: 1.22 Link to plots, accessed 9 June 2022
TEST_2_3 (Straight line crossings with stopping, re-calibration, constant orientation) maxVelocity_translation [mm/s]: 200 maxAcceleration_translation [mm/s${}^{2}$]: 500 maxVelocity_rotation [deg/s]: — maxAcceleration_rotation [deg/s${}^{2}$]: —	Max. translation error [mm]: 32.92 Avg. translation error [mm]: 6.92 Max. orientation error [deg]: 10.97 Avg. orientation error [deg]: 0.92 Link to plots, accessed 9 June 2022
TEST_3_1 (General motion—Hand Guidance trajectory planning)	Max. translation error [mm]: 113.78 Avg. translation error [mm]: 12.20 Max. orientation error [deg]: 13.82 Avg. orientation error [deg]: 2.11 Link to plots, accessed 9 June 2022
TEST_3_2 (General motion—Hand Guidance trajectory planning, 1/3 of the previous velocity)	Max. translation error [mm]: 24.26 Avg. translation error [mm]: 5.26 Max. orientation error [deg]: 2.49 Avg. orientation error [deg]: 0.57 Link to plots, accessed 9 June 2022

## Appendix B

Links to supplementary video materials:

- **Motion data post-processing**—effect of oscillatory smoothing filter dynamics:
  Link: Movie 1, accessed 9 June 2022
- **Pilot application**—tracking system for robot-assisted spray painting:
  Link: Movie 2, accessed 9 June 2022
- **Alternate mirror link:**
  Link, accessed 9 June 2022
